# Striatal Dopamine D_2_/D_3_ Receptor Availability Is Associated with Executive Function in Healthy Controls but Not Methamphetamine Users

**DOI:** 10.1371/journal.pone.0143510

**Published:** 2015-12-14

**Authors:** Michael E. Ballard, Andy C. Dean, Mark A. Mandelkern, Edythe D. London

**Affiliations:** 1 Department of Psychiatry and Biobehavioral Sciences, University of California Los Angeles, Los Angeles, California, United States of America; 2 VA Greater Los Angeles Healthcare System, Los Angeles, California, United States of America; 3 Brain Research Institute, University of California Los Angeles, Los Angeles, California, United States of America; 4 Department of Physics, University of California Irvine, Irvine, California, United States of America; 5 Department of Molecular and Medical Pharmacology, University of California Los Angeles, Los Angeles, California, United States of America; University of Western Ontario, CANADA

## Abstract

**Background:**

Dopamine D_2_/D_3_ receptor availability in the striatum has been linked with executive function in healthy individuals, and is below control levels among drug addicts, possibly contributing to diminished executive function in the latter group. This study tested for an association of striatal D_2_/D_3_ receptor availability with a measure of executive function among research participants who met DSM-IV criteria for methamphetamine dependence.

**Methods:**

Methamphetamine users and non-user controls (*n* = 18 per group) completed the Wisconsin Card Sorting Test and positron emission tomography with [^18^F]fallypride.

**Results:**

The methamphetamine users displayed significantly lower striatal D_2_/D_3_ receptor availability on average than controls after controlling for age and education (*p* = 0.008), but they did not register greater proportions of either perseverative or non-perseverative errors when controlling for education (both *p*s ≥ 0.622). The proportion of non-perseverative, but not perseverative, errors was negatively correlated with striatal D_2_/D_3_ receptor availability among controls (*r* = -0.588, *p* = 0.010), but not methamphetamine users (*r* = 0.281, *p* = 0.258), and the group-wise interaction was significant (*p* = 0.030).

**Conclusions:**

These results suggest that cognitive flexibility, as measured by perseverative errors on the Wisconsin Card Sorting Test, is not determined by signaling through striatal D_2_/D_3_ receptors in healthy controls, and that in stimulant abusers, who have lower D_2_/D_3_ receptor availability, compensation can effectively maintain other executive functions, which are associated with D_2_/D_3_ receptor signaling in controls.

## Introduction

Drug addiction is a complex disorder that often is associated with inflexible behavior and deficient executive function [1]. Numerous studies have demonstrated poorer performance on tasks that assess executive function among individuals with a history of chronic drug abuse relative to healthy controls [2–14]. Problems with executive function, including cognitive inflexibility, difficulty focusing attention, and diminished self-control, have been implicated in the etiology of compulsive drug use, and reduced function in these domains has been viewed as a substantial impediment to successful treatment (for reviews, see [1, 15, 16]). As such, research aimed at elucidating the neural underpinnings of the relatively less effective executive function among some substance-dependent individuals compared to non-user controls may lead to new therapeutic interventions.

Executive function is primarily subserved by the prefrontal cortex, which exerts “top-down” control over subcortical structures, such as the striatum and amygdala, which are responsive to “bottom-up” sensory input [17]. Despite being principally ascribed to the prefrontal cortex [17–19], however, the processes of decision-making and selection of actions are mediated by subcortical structures as well. In particular, D_2_/D_3_ receptor availability in the striatum has been found to be correlated positively with performance on laboratory tests that rely heavily on executive processes in healthy individuals [20–25], and in carriers of the Huntington’s disease mutation [26]. This is particularly relevant to addictive disorders because striatal D_2_/D_3_ receptor availability is typically lower among drug addicts than non-user controls (for a review, see [27]). As such, it stands to reason that low striatal D_2_/D_3_ receptor availability may be linked with poor performance on laboratory tests of executive function among individuals with substance-use disorders. Yet, to our knowledge, no studies have examined whether the relationships between striatal D_2_/D_3_ receptor availability and executive functions seen in healthy individuals extend to substance abusers, who have relatively low striatal D_2_/D_3_ receptor availability.

In this study, striatal D_2_/D_3_ receptor availability was examined in relation to performance on the Wisconsin Card Sorting Test (WCST), a laboratory measure of executive function. Research participants who met DSM-IV criteria for methamphetamine (MA) dependence were compared with a group of healthy controls. MA-dependent individuals were selected as a group for study because case-control studies find that they display low striatal D_2_/D_3_ receptor availability (for a review, see [28]) and poorer performance on the WCST than healthy controls ([2, 3]; although several studies have not observed similar performance differences among cocaine users, e.g. [4–6, 12, 29, 30]). On the basis of the literature cited above, we expected that MA users would exhibit lower striatal D_2_/D_3_ receptor availability and worse performance on the WCST. We hypothesized that striatal D_2_/D_3_ receptor availability would be negatively correlated with errors on the WCST among both MA users and non-users. Further, we hypothesized that WCST performance measures would be more strongly related to striatal BP_ND_ in MA users than in controls, in line with our previous finding regarding the relationship between striatal BP_ND_ and temporal discounting, another measure of executive function [31].

## Methods

### Participants

Procedures were approved by the University of California Los Angeles (UCLA) Office for Protection of Research Subjects. Participants were recruited using Internet and local newspaper advertisements. All provided written informed consent and underwent eligibility screening using questionnaires, the Structured Clinical Interview for DSM-IV (SCID) [32], and a physical examination. Eighteen individuals who met criteria for current MA dependence, but were not seeking treatment for their addiction, and 18 controls, completed the study. D_2_/D_3_ receptor-availability data from all but two of the MA users and all of the controls have been reported previously [31], and smaller subsets were included in other studies from our laboratory regarding striatal D_2_/D_3_ receptor availability [33–37].

The exclusion criteria were: CNS, cardiovascular, pulmonary, hepatic, or systemic disease; HIV seropositive status; pregnancy; lack of English fluency; MRI ineligibility (e.g. metal implants, claustrophobia); current use of psychotropic medications; current Axis I disorder including substance abuse or dependence for any substance other than nicotine (all MA users met criteria for MA dependence; substance-induced mood disorders were also not exclusionary for this group).

A diagnosis of MA dependence and a positive urine test for MA metabolites at intake were required for MA-group participants, who completed the study as inpatients at the UCLA General Clinical Research Center, and were prohibited from using any drugs (besides nicotine in cigarettes and caffeine in beverages) for 4–7 days before testing. Most MA users completed the behavioral and imaging measures 2 days apart (*n*s = 16 within 1 week, one 11 days apart, and one 415 days apart). Controls were studied on a nonresidential basis, and most completed the measures within a few days or weeks (*n*s = seven within 1 week, nine 1–6 weeks apart, one 337 days apart, and one 488 days apart). Relationships between imaging and behavioral variables did not depend on the lag between measures, and excluding the individuals with long lags did not substantially change the results. Each participant was required to provide a urine sample on each test day that was negative for amphetamine, cocaine, MA, benzodiazepine, opiate, and cannabinoid metabolites. Compensation was provided in the form of cash, gift certificates, and vouchers.

### Executive function

Executive function was assessed with the Wisconsin Card Sorting Test (WCST) [38]. While performing this non-computerized version of the test, the participant is presented with four sample cards, each depicting between one and four geometric shapes (triangle, star, cross, or circle), all of the same color (red, green, yellow, or blue). He or she is instructed to select a new card on which the items are of a different number, shape, and/or color, and then to match the drawn card to one of the sample cards using one of the attributes, without knowing the pre-defined matching criterion. The participant is informed after each response whether he/she was correct or incorrect. Subsequently, a new card is drawn, and the task proceeds. Once 10 consecutive correct matches are made, the criterion for success is switched (e.g., where matching by color first yields success, the matching criterion is switched to geometric shape, etc.). The task is untimed, and terminates when the participant reaches six correct categories or cycles through all 128 cards. The WCST is widely used as a test of executive function, and studies have shown that individuals with a history of MA abuse often perform more poorly on this task than non-user controls (e.g. users tend to register more errors) [2, 3]. In this study, main outcome measures of interest included the proportion of trials registered as perseverative errors (i.e., continuing to match on an attribute that was previously identified as incorrect within a given matching rule) and the proportion of trials registered as non-perseverative errors (i.e., incorrectly matching in a manner not previously established as incorrect). Although the number of correct categories can be tallied (number of times a set of 10 cards were correct in a row for the same attribute), we did not examine this variable in relation to receptor availability because only three participants (all MA users) achieved less than the maximum six categories possible.

### D_2_/D_3_ receptor availability

Dopamine D_2_/D_3_ receptor availability was assessed using a Siemens EXACT HR+ PET scanner in 3D mode with [^18^F]fallypride as the radioligand [39]. Following a 7-min transmission scan acquired using a rotating ^68^Ge/^68^Ga rod source to measure and correct for attenuation, PET dynamic data acquisition was initiated with a bolus injection of [^18^F]fallypride (~5 mCi ± 5%, specific activity ≥ 1 Ci/μmol). Emission data were acquired in two 80-min blocks, separated by a 10-20-min break.

Raw PET data were corrected for decay, attenuation, and scatter, and then reconstructed using ordered-subsets expectation-maximization (OSEM) (3 iterations; 16 subsets), using ECAT v7.2 software (CTI PET Systems Inc., Knoxville, TN). Reconstructed data were combined into 16 images (each representing an average of 10 min of dynamic data), and the images were motion-corrected using FSL McFLIRT [40], and co-registered to the individual’s structural MRI scan image using a six-parameter, rigid-body transformation computed with the ART software package [41]. Structural images were magnetization-prepared, rapid-acquisition, gradient-echo (MPRAGE) scans, acquired during a separate session using a Siemens Sonata 1.5T MRI scanner. All images were registered to MNI152 space using FSL FLIRT [42]. The primary volume of interest (VOI) was the striatum, and exploratory analyses were carried out in other subcortical and cortical VOIs with appreciable [^18^F]fallypride BP_ND_ (i.e., globus pallidus, amygdala, thalamus, midbrain, insula, hippocampus, anterior cingulate cortex, and medial and lateral orbitofrontal cortices) in order to explore the specificity of potential relationships observed in the striatum. Volumes of interest (VOIs) were derived from the Harvard-Oxford atlases transformed into individual native space, or defined using FSL FIRST [41]. For VOI analysis, the striatum was divided into three functional subdivisions as described previously [43]: the limbic striatum consisted of the ventral striatum; the associative striatum consisted of the precommissural dorsal putamen, precommissural dorsal caudate, and postcommissural caudate; and the sensorimotor striatum consisted of the postcommissural putamen [44].

Time-activity data within VOIs were imported into the PMOD 3.2 kinetic modeling analysis program (PKIN; PMOD Technologies Ltd., Zurich, Switzerland), and time-activity curves were fit using the Simplified Reference Tissue Model 2, SRTM2 [45]. The cerebellum (excluding the vermis) was used as the reference region [46]. The rate parameter for transfer of the tracer from the reference region to plasma (*k*_2_′) was computed as the volume-weighted average of estimates from fits to receptor-rich regions (caudate and putamen) calculated using the simplified reference tissue model (SRTM) [47], as suggested by Ichise et al. [48]. Time-activity curves were re-fit using SRTM2 [45], with the computed *k*_2_′ value applied to fits to all brain regions. Regional binding potential referred to non-displaceable binding and was calculated as BP_ND_ = *R*_1_(*k*_2_′ / *k*_2a_ - 1), where *R*_1_ = *K*_1_ / *K*_1_′ is the ratio of tracer-delivery parameters for the tissue of interest and reference tissue, *k*_2_′ is the rate parameter for the transfer of tracer from the reference tissue to the plasma, and *k*_2a_ is the effective rate parameter for transfer of tracer from the tissue of interest to the plasma [49–51]. Volume-weighted bilateral averages of all VOIs were used for analyses.

### Statistical analyses

Statistical analyses were carried out using SPSS v23 (IBM Corp., Armonk, NY). Continuous variables were assessed for homogeneity of variance across groups using Levene’s tests. Demographic variables were examined for group differences using two-tailed independent samples *t*-tests, Mann-Whitney *U*-tests, or Fisher’s exact tests, as appropriate. Group differences in executive function measures and BP_ND_ were tested using separate independent-samples *t*-tests, and ANOVA was used to confirm group differences when controlling for confounding demographic variables; covariates were identified using forward regression predicting the dependent measure of interest. Linear regressions were used to test potential relationships between putative predictor variables and outcome measures of interest, with potential confounding demographic variables included as covariates. Potential group differences in the strength of the relationships between BP_ND_ and WCST measures were assessed using linear regression, with a group x BP_ND_ interaction term entered in the model along with group and BP_ND_; interactions with covariates were not included in the model. The threshold for statistical significance was set at α = 0.05 for all analyses.

## Results

The groups included similar proportions of males and females (*p* = 1.00; Table 1), as well as tobacco smokers and non-smokers (*p* = 0.471), and did not differ in age (*p* = 0.258) or ethnic group composition (*p* = 0.572); however, MA users reported significantly fewer years of formal education than controls on average (*p* = 0.010).

**Table 1:** Characteristics of research participants.

Group	Controls (*n* = 18)	MA users (*n* = 18)
Sex (M/F)	11/7	10/8
Age (years)	36.4 ± 9.2 (19–51)	33.1 ± 7.8 (19–46)
Education (years)	14.3 ± 2.2 (10–18)	12.6 ± 1.5 (11–16)*
Ethnicity (White/Hispanic or Latino/Asian/Native American/Other^†^)	11/4/2/1/0	11/4/2/0/1
No. daily tobacco smokers (M/F)	7/4	9/5
Cigarettes per day (daily smokers only)	12.8 ± 4.9 (8–20)	12.1 ± 11.7 (3–40)
Years smoking (daily smokers only)	17.5 ± 11.4 (3–35)	17.3 ± 9.6 (3–34)
FTND score (daily smokers only)	3.5 ± 2.3 (0–8)	3.1 ± 2.8 (0–9)
Duration of regular MA use (years)	N/A	9.3 ± 7.9 (0.5–24)
Frequency of MA use (days in last 30 days)	N/A	21.9 ± 8.7 (5–30)
Intensity of MA use (grams in last week)	N/A	2.8 ± 3.3 (0.3–14.5)

Data are presented as mean + SD (range), except for sex, ethnicity, and smoking status.

^†^Other refers to individuals not identifying as White, African American, Hispanic/Latino, Asian, or Native American

FTND: Fagerström Test for Nicotine Dependence (possible range: 0 [low]—10 [high]; [52])

*Significant group difference, p < .05.

As shown in Table 2, striatal BP_ND_ was lower among MA users than controls, and this group difference was statistically significant when controlling for both age and years of formal education; age and education were respectively selected first and second (while sex and smoking status were excluded) by a forward step-wise regression predicting striatal BP_ND_ with demographic variables (correlation between striatal BP_ND_ and age: *r* = -0.637, *p* < 0.0005; correlation between striatal BP_ND_ and education: *r* = 0.418, *p* = 0.011; S1 and S2 Tables), consistent with previous reports [53, 54].

**Table 2:** Comparison of striatal BP_ND_ and WCST performance between MA users and controls (*n* = 18 each).

Measure	Means (SEM)		Independent samples *t*-test		ANOVA with covariates		
	MA users	Controls	*t* (df)	*p*	*F* (df)	*p*	*η*_*p*_^2^
Whole striatum BP_ND_	17.56 (0.64)	19.85 (0.93)	2.03 (34)	0.050	9.49 (1,32)	0.004	0.229
LST BP_ND_	15.53 (0.54)	17.45 (0.73)	2.12 (34)	0.042	10.26 (1,32)	0.003	0.243
AST BP_ND_	17.41 (0.64)	19.45 (0.95)	1.78 (34)	0.085	7.11 (1,32)	0.012	0.182
SMST BP_ND_	19.44 (0.69)	21.76 (1.06)	1.84 (34)	0.075	9.14 (1,32)	0.005	0.222
WCST proportion of							
NPEs	0.10 (0.02)	0.08 (0.01)	1.61 (34)	0.117	0.34 (1,33)	0.566	0.010
PEs	0.12 (0.02)	0.09 (0.01)	1.36 (34)	0.184	0.29 (1,33)	0.592	0.009

ANOVA covariates are age and years of formal education for BPND analyses, and age for WCST analyses

LST: limbic striatum; AST: associative striatum; SMST: sensorimotor striatum;

NPEs: non-perseverative errors; PEs: perseverative errors.

Compared to controls, MA users tended to register greater proportions of both non-perseverative errors and perseverative errors, but there was no evidence of group differences in the proportions of errors when controlling for years of formal education; education was selected as the sole predictor (while age, sex, years of education, and smoking status were excluded) by a forward step-wise regression predicting WCST proportion of non-perseverative errors with demographic variables (correlation between the proportion of non-perseverative errors and education: *r* = -0.435, *p* = 0.008; S1 and S3 Tables).

Multiple regression analyses indicated that the relationship between the proportion of non-perseverative errors and striatal BP_ND_ differed significantly between MA users and controls, as evidenced by the significant effect of a group x BP_ND_ product term, which was calculated to represent the interaction effect and entered into the model along with group and BP_ND_ (*R* ^2^ = 0.13, *F*_1,32_ = 5.13, *p* = 0.030). Analogous post-hoc analyses indicated that the group by striatal BP_ND_ interaction was apparent in the associative subdivision of the striatum (group x BP_ND_: *R* ^2^ = 0.22, *F*_1,32_ = 5.39, *p* = 0.027, with apparently smaller effects in limbic [group x limbic striatum BP_ND_: *p* = 0.143] and sensorimotor divisions of the striatum [group x sensorimotor striatum BP_ND_: *p* = 0.078]), but this effect was not significant following correction for multiple comparisons using the Holm-Bonferroni method.

**Fig 1:**
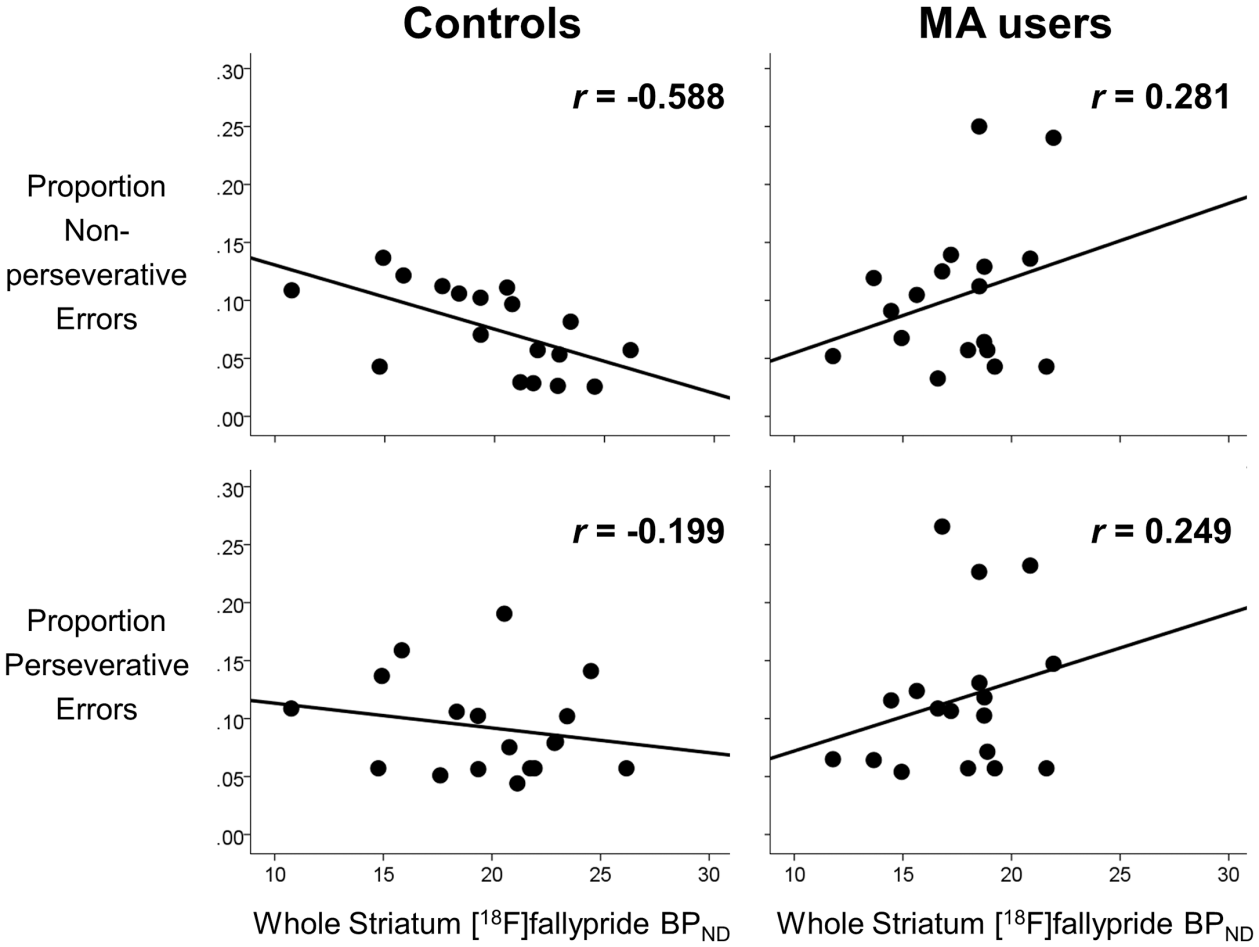
Relationships between striatal D_2_/D_3_ receptor availability and executive function measures. Regression lines illustrate correlations between striatal dopamine D_2_/D_3_ receptor availability (indexed by [^18^F]fallypride BP_ND_) and proportions of trials registered as non-perseverative (top row) and perseverative (bottom row) errors in methamphetamine (MA) users and non-user controls. Pearson product-moment correlation coefficients are shown (*r* values).

**Fig 2:**
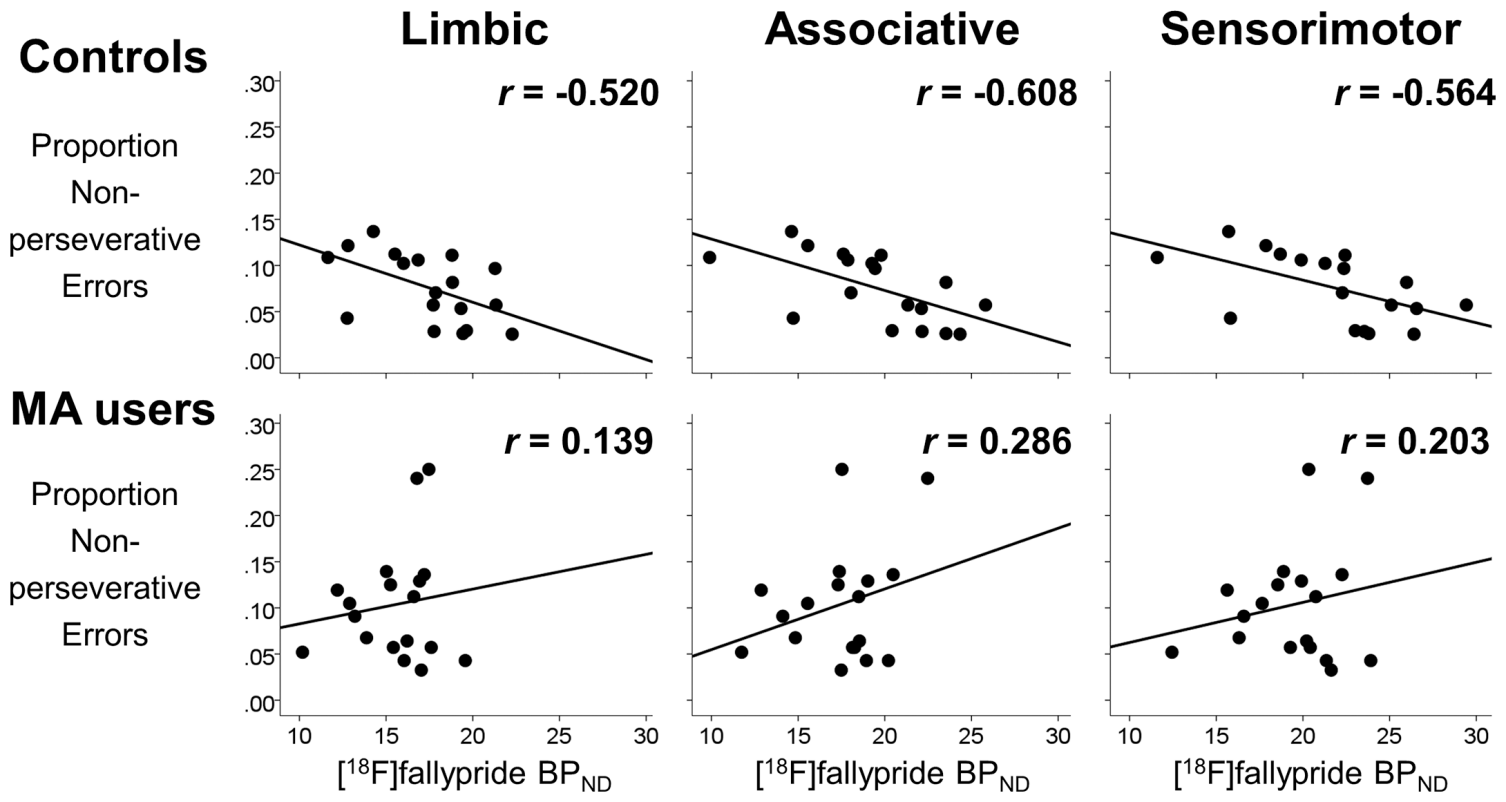
Relationships between D_2_/D_3_ receptor availability in striatal subregions and executive function measures. Regression lines illustrate correlations between striatal dopamine D_2_/D_3_ receptor availability (indexed by [^18^F]fallypride BP_ND_) and proportions of trials registered as non-perseverative and perseverative errors in the striatal functional subdivisions of methamphetamine (MA) users (bottom row) and non-user controls (top row). Pearson product-moment correlation coefficients are shown (*r* values).

A forward step-wise regression selected striatal BP_ND_ (while excluding age, sex, education, and smoker status) as the best and sole predictor of the proportion of non-perseverative errors among controls, accounting for 35% of the variance across this group (*F*_1,16_ = 8.45, *p* = 0.010). The correlation coefficient for the relationship between the proportion of non-perseverative errors and striatal BP_ND_ among controls was *r* = -0.588 (MA users: *r* = 0.281, *p* = 0.258; Fig 1). Analogous post-hoc analyses revealed significant negative correlations between the proportion of non-perseverative errors and BP_ND_ in all three striatal functional subdivisions among controls (limbic striatum: *r* = -0.520, *p* = 0.027; associative striatum: *r* = -0.608, *p* = 0.007; sensorimotor striatum: *r* = -0.564, *p* = 0.015; Fig 2), and these survived correction for multiple comparisons using the Holm-Bonferroni method. An analogous forward step-wise regression in MA users did not select any of the independent variables (i.e., striatal BP_ND_, age, sex, education, smoker status) as significant predictors of the proportion of non-perseverative errors in that group.

With respect to perseverative errors, there was no evidence of a group by striatal BP_ND_ interaction (*p* = 0.183), and the proportion of perseverative errors was not significantly correlated with striatal BP_ND_ in either group, (both *p* ≥ 0.318; Fig 1). Although the proportions of perseverative and non-perseverative errors were positively correlated in both groups (controls: *r* = 0.519, *p* = 0.027; MA users: *r* = 0.646, *p* = 0.004), a post hoc Steiger’s *z*-test indicated that, among controls only, striatal BP_ND_ was significantly more strongly correlated with the proportion of non-perseverative than perseverative errors (*z* = -1.79, one-tailed *p* = 0.037).

## Discussion

The goal of this study was to test the hypotheses that striatal D_2_/D_3_ receptor availability would be linked with executive function, as measured by the WCST, with greater receptor availability accompanying better performance; and that this association would be stronger in MA-dependent participants than in healthy controls. This hypothesis was based mainly on reports that striatal D_2_/D_3_ receptor availability is positively associated with performance on laboratory tests of executive function among healthy individuals who do not use drugs of abuse [20–25], and is typically lower among drug addicts than non-user controls (for a review, see [27]). We reasoned that the linear relationship found among non-users would extend to, and potentially would be stronger among MA-users, in a manner analogous to the greater dependence on D_2_/D_3_ receptor availability of performance on a delay discounting task in MA-dependent than in control subjects [31]. In contrast to our hypothesis and to previous reports [2, 3], we found that MA users did not register greater proportions of errors in this study, when controlling for education, despite displaying significantly lower striatal D_2_/D_3_ receptor availability on average than controls, after controlling for age and education. Furthermore, striatal D_2_/D_3_ receptor availability was negatively correlated with the proportion of non-perseverative errors, but not perseverative errors among controls but not MA users.

The present study is one of the first to examine a discrete measure of executive function in relation to D_2_/D_3_ receptor availability in individuals with a substance-use disorder [55]; although a small number have utilized alternate neurochemical measures of striatal dopaminergic neurotransmission [56, 57]. Thus, it addresses an important gap in the literature because executive deficits, particularly involving cognitive flexibility and sensitivity to negative feedback, have been implicated in the etiology of compulsive drug use, and are viewed as substantial obstacles to treatment (for reviews, see [1, 15, 16]). Our results suggest that, although certain aspects of cognitive function, indexed by non-perseverative errors on the WCST, may be related to striatal D_2_/D_3_ receptor availability in individuals who do not abuse drugs, low striatal D_2_/D_3_ receptor availability does not appear to influence one measure of executive function, specifically cognitive inflexibility, as measured by perseverative errors among MA users. This finding is surprising in light of findings that acute administration of a D_2_ receptor antagonist [58] as well as administration of MA according to a subchronic regimen that reduces striatal neurochemical markers of dopaminergic neurotransmission [59] impair cognitive flexibility in monkeys. The present finding in humans is intriguing because it raises the possibility that compensatory neural mechanisms serve to buffer at least some aspects of executive function from the potentially detrimental effects of low striatal D_2_/D_3_ receptor availability in stimulant abusers.

That striatal D_2_/D_3_ receptor availability was negatively correlated with the proportion of non-perseverative, but not perseverative, errors among healthy controls is broadly consistent with a report that dorsal striatal D_2_/D_3_ receptor availability is positively associated with sensitivity to positive feedback (i.e., the tendency to follow positive feedback with a correct response) but not to negative feedback (i.e., the ability to change matching criteria following negative feedback) in healthy monkeys during reversal learning [60]. An important distinction, however, is that although a high proportion of perseverative errors in the WCST essentially reflects insensitivity to negative feedback (the tendency to persist with a matching criterion that the subject has already been informed is incorrect), the proportion of non-perseverative errors is not a pure measure of insensitivity to positive feedback—i.e., it could reflect imprecise learning of the matching criterion, or deficits in working memory, attention, and other cognitive processes. Although the number of perseverative errors on the WCST essentially reflects insensitivity to negative feedback, it may not constitute a direct measure of negative reinforcement learning in the context of the larger task, which requires greater executive control than simpler reinforcement learning tasks. Indeed, in contrast to a recent PET study in humans, which found an inverted U-shaped relationship between striatal D_2_/D_3_ receptor availability and a more pure measure of learning from negative feedback [61], the present study found no such relationship between striatal D_2_/D_3_ receptor availability and the proportion of perseverative errors on the WCST, either in the combined sample (*p* = 0.567), or in controls (*p* = 0.733) or MA users (*p* = 0.502), separately.

Still, current computational theories hypothesize that learning from negative feedback is mediated, in part, by D_2_ receptor-containing striatal medium spiny neurons in the indirect (or ‘no-go’) pathway [62], and that reductions in striatal D_2_/D_3_ receptor levels, which may occur following chronic methamphetamine abuse [59], would serve to erode dopaminergic control over the ‘no-go’ system, thus diminishing the capacity to learn from negative feedback. Also in line with a function of D_2_/D_3_ receptor signaling in learning form negative feedback is the observation that carriers of the A1 allele of the *ANKK1*-Taq1A polymorphism exhibit subtle performance deficits in reversal learning as compared to homozygotes without this allele [63] and show reduced interaction between the posterior medial frontal cortex and the hippocampus—referred to as the performance monitoring network—in response to negative feedback than those without the A1 allele [64]. As well, results from a study involving a small sample of abstinent cocaine abusers and non-user controls who performed the color-word Stroop task, which measures executive function and is heavily dependent on cognitive flexibility, provide preliminary evidence that lower striatal D_2_/D_3_ receptor availability is linked to blunted neural responses (e.g., midbrain activation) during error processing among stimulant abusers [55].

Contrary to this reasoning, MA users did not register greater proportions of perseverative errors than controls, despite having lower striatal D_2_/D_3_ receptor availability on average. However, it has also been reasoned that under conditions of low tonic dopaminergic transmission, which is hypothesized to coincide with reduced striatal D_2_/D_3_ receptor density following chronic stimulant abuse [27], the capacity to learn from negative feedback is enhanced [61, 62], which could help to buffer MA users from the potentially detrimental effects of low striatal D_2_/D_3_ receptor density on learning from negative feedback.

In the single other study that examined striatal D_2_/D_3_ receptor availability in relation to WCST performance, the total number of errors, a raw measure that does not take into account the type of error or total number of trials, was negatively correlated with D_2_/D_3_ receptor availability in both striatal regions tested (caudate and putamen) of healthy control subjects [20]; a similar trend was found in controls studied here (*r* = -0.405, *p* = 0.091).

That MA users did not register significantly greater proportions of perseverative or non-perseverative errors than controls is consistent with two previous studies [56, 65]. Two others found significant group differences with both perseverative and non-perseverative errors on the WCST: one with MA users abstinent longer than one month [3]; the other with MA users abstinent on average longer than two years [2]. Potential reasons for this discrepancy between the results of these latter two studies and the lack of a difference in performance compared with controls in the present study are not immediately clear, but may reflect a variety of differences in the samples studied. For example, in one of the two prior studies that found a group difference in WCST performance, all of the MA users (*N* = 32) self-administered the drug by the intravenous route and were Asian, and HIV seropositive status was not excluded [2], unlike the characteristics of the sample studied here. In the other study that found differences in WCST performance in a sample of abstinent MA users, all MA users (*N* = 43) were hospitalized treatment-seeking patients [3], also unlike the participants in the present study. Certainly, the larger sample sizes in those studies provided more power than those of the present study to detect group differences; however given the exceedingly marginal group difference in WCST performance seen here after controlling for education, it seems unlikely that using similar-sized samples in this study would have returned a significant group difference in WCST performance. Indeed, several studies have also found no significant difference on measures of WCST errors between controls and other populations of drug abusers [9, 66], who also typically display low striatal D_2_/D_3_ receptor availability (for a review, see [27]), including stimulant users [4–6, 12, 29, 30].

That WCST error rates can be similar between controls and MA users despite the latter group displaying lower striatal D_2_/D_3_ receptor availability, coupled with the finding that the non-perseverative error rate is correlated with striatal D_2_/D_3_ receptor availability in controls but not MA users, suggests that striatal D_2_/D_3_ receptor availability contributes to certain aspects of executive function in healthy individuals, but that these associations become uncoupled in MA dependence. As such, executive function may be primarily subserved by non-D_2_/D_3_ mechanisms in chronic stimulant abusers who have low striatal D_2_/D_3_ receptor availability. Broadly in line with this view is the observation that a deficit in reversal learning in monkeys, induced by an escalating dose regimen of MA that produced persistent reductions in striatal D_2_/D_3_ receptor availability, was transient whereas the neurochemical loss was long-term [59].

It is important to note that both WCST performance measures and D_2_/D_3_ receptor availability were significantly correlated with years of formal education in our sample. This collinearity in predictive variables is an important limitation because it makes it difficult to disentangle the influence of education on the relationship between WCST performance and striatal D_2_/D_3_ receptor availability. Despite the established relationship between cognition and education, studies of cognition in MA users do not consistently control for education [67]. Studies testing the association of D_2_/D_3_ receptor availability with cognition have also not consistently considered the potential confounding effect of education [21, 24–26]. Still, considering the maladaptive behavioral patterns associated with low striatal D_2_/D_3_ receptor availability (for a review, see [27]), it seems reasonable to expect that low striatal D_2_/D_3_ receptor availability may contribute to less educational attainment. Another possibility is that lack of school-related enrichment could result in low D_2_/D_3_ receptor availability, but this is not known. That only a subset of participants completed a measure of IQ is also a notable limitation, because it would have helped to indicate whether the relationship found with WCST non-perseverative errors is specific to the cognitive domains involved, or whether this finding might be better characterized as being related to general cognition. Future studies will help to determine the extent to which the relationship depends on educational attainment and IQ.

Evidence from previous PET studies indicates that the link between WCST performance and D_2_/D_3_ receptor availability in healthy adults is not limited to the striatum. Two of these studies showed that total perseverative errors were correlated negatively with D_2_/D_3_ receptor availability in the hippocampus [68, 69]; and another, which surveyed several extrastriatal regions found positive correlations between perseverative as well as non-perseverative errors and D_2_/D_3_ receptor availability in the right anterior cingulate cortex only [70]. Broadly in line with the former studies, we detected a modest negative association between perseverative error rate and D_2_/D_3_ receptor availability in the hippocampi of control subjects (*r* = -0.327, *p* = 0.186); but in contrast with the latter study, we saw no evidence of a positive association between D_2_/D_3_ receptor availability in the anterior cingulate cortex and the rate of either perseverative (*r* = -0.185, *p* = 0.462) or non-perseverative errors (*r* = -0.174, *p* = 0.490). In fact, exploratory correlational analyses did not return a *p*-value less than 0.05 with respect to the relationships between either error measure and D_2_/D_3_ receptor availability in any of the cortical or extrastriatal subcortical regions tested (S4 Table); although they revealed some evidence of potential negative relationships between non-perseverative error rate and D_2_/D_3_ receptor availability in the globus pallidus and thalamus among controls. The suggestion from the exploratory correlation analyses that the relationship between non-perseverative error rate and D_2_/D_3_ receptor availability is primarily restricted to the striatum is also noteworthy because it contrasts with evidence that temporal discounting of rewards (which also depends on executive functions) is associated with D_2_/D_3_ receptor availability in a number of extrastriatal regions in addition to the striatum [31].

In summary, we found that MA users displayed significantly lower striatal D_2_/D_3_ receptor availability on average than controls after controlling for age and education, but they did not register greater proportions of perseverative or non-perseverative errors on the WCST when controlling for education. The proportion of non-perseverative errors, but not perseverative errors, was negatively correlated with striatal D_2_/D_3_ receptor availability among controls, but not MA users. Taken together, these results suggest that non-D_2_/D_3_ receptor-mediated mechanisms can effectively buffer some aspects of executive function from deficient striatal D_2_/D_3_ receptor-mediated neurotransmission in chronic stimulant abusers.

## Supporting Information

S1 Data (XLSX)

S1 Table Correlations of the variables (*n* = 36). (PDF)

S2 Table Forward step-wise regression results with whole striatum BP_ND_ as the dependent variable, and demographic variables as predictors. (PDF)

S3 Table Forward step-wise regression results with WCST proportion of non-perseverative errors as the dependent variable, and demographic variables as predictors. (PDF)

S4 Table Exploratory tests of relationships between D2/D3 receptor availability in extrastriatal regions and executive function measures. (PDF)
