# Supplementary material for: Striatal Dopamine D2/D3 Receptor Availability Is Associated with Executive Function in Healthy Controls but Not Methamphetamine Users
Source: PLoS One. 2015 Dec 14;10(12):e0143510. doi: 10.1371/journal.pone.0143510 (PMC4699455; doi:10.1371/journal.pone.0143510)
Supplement: S1 Table — (PDF) [file pone.0143510.s002.pdf]

S1 Table. Correlations of the variables (*n* = 36)

	2	3	4	5	6	7
1. Whole striatum BP_{ND}	-.186	-.047	-.637*	.398*	.418*	-.478*
2. WCST proportion of NPEs		.633*	.170	-.005	-.435*	.292
3. WCST proportion of PEs			-.106	.173	-.347*	.061
4. Age				-.480*	.024	.343*
5. Sex					.159	-.173
6. Years of education						-.190
7. Smoker status						

NPEs: non-perseverative errors; PEs: perseverative errors

Sex coded as 0 = male, 1 = female

Smoker status coded as 0 = non-smoker, 1 = smoker

*$p < 0.05$ (2-tailed)
