# Supplementary material for: Striatal Dopamine D2/D3 Receptor Availability Is Associated with Executive Function in Healthy Controls but Not Methamphetamine Users
Source: PLoS One. 2015 Dec 14;10(12):e0143510. doi: 10.1371/journal.pone.0143510 (PMC4699455; doi:10.1371/journal.pone.0143510)
Supplement: S2 Table — (PDF) [file pone.0143510.s003.pdf]

S2 Table. Forward step-wise regression results with whole striatum BP_{ND} as the dependent variable, and demographic variables as predictors

	b	*SE-b*	Beta	Pearson *r*	sr^2	Structure Coefficient
Constant	17.992	2.994				
Age*	-.265	.460	-.647	-.637	.419	-.827
Years of education*	.741	.190	.433	.418	.187	.543

R^2 = .593, Adjusted R^2 = .568

sr^2 is the squared semi-partial correlation

*p <0.05
