# Supplementary material for: Striatal Dopamine D2/D3 Receptor Availability Is Associated with Executive Function in Healthy Controls but Not Methamphetamine Users
Source: PLoS One. 2015 Dec 14;10(12):e0143510. doi: 10.1371/journal.pone.0143510 (PMC4699455; doi:10.1371/journal.pone.0143510)
Supplement: S3 Table — (PDF) [file pone.0143510.s004.pdf]

S3 Table. Forward step-wise regression results with WCST proportion of non-perseverative errors as the dependent variable, and demographic variables as predictors

	b	*SE-b*	Beta (Pearson *r*)	Structure Coefficient
Constant	.238	.053		
Years of education*	-.011	.004	-.435	-2.302

R^2 = .189, Adjusted R^2 = .165

*p <0.05
