# Supplementary material for: Striatal Dopamine D2/D3 Receptor Availability Is Associated with Executive Function in Healthy Controls but Not Methamphetamine Users
Source: PLoS One. 2015 Dec 14;10(12):e0143510. doi: 10.1371/journal.pone.0143510 (PMC4699455; doi:10.1371/journal.pone.0143510)
Supplement: S4 Table — (PDF) [file pone.0143510.s005.pdf]

S4 Table. Exploratory tests of relationships between D_2/D_3 receptor availability in extrastriatal regions and executive function measures

	Controls (*n* = 18)		MA users (*n* = 18)	
	Non-perseverative Error Rate	Perseverative Error Rate	Non-perseverative Error Rate	Perseverative Error Rate
Globus pallidus	-0.468 (0.050)	-0.223 (0.373)	0.148 (0.557)	0.272 (0.275)
Amygdala	-0.334 (0.176)	-0.274 (0.271)	0.164 (0.515)	0.164 (0.515)
Thalamus	-0.456 (0.057)	-0.172 (0.495)	0.394 (0.106)	0.278 (0.264)
Midbrain	-0.344 (0.162)	-0.038 (0.882)	0.385 (0.115)	0.351 (0.153)
Insula	-0.143 (0.571)	-0.060 (0.814)	0.011 (0.967)	0.146 (0.563)
Hippocampus	-0.386 (0.114)	-0.327 (0.186)	-0.009 (0.972)	0.149 (0.556)
ACC	-0.174 (0.490)	-0.185 (0.462)	0.199 (0.428)	0.158 (0.531)
mOFC	-0.186 (0.461)	-0.135 (0.594)	0.083 (0.743)	0.195 (0.438)
lOFC	-0.216 (0.389)	-0.076 (0.766)	0.139 (0.581)	0.308 (0.213)

Data are presented as Pearson correlation coefficients (*p* value; uncorrected).
ACC: anterior cingulate cortex; *mOFC*: medial orbitofrontal cortex;
lOFC: lateral orbitofrontal cortex
